# Artificial Light Increases Nighttime Prevalence of Predatory Fishes, Altering Community Composition on Coral Reefs

**DOI:** 10.1111/gcb.70002

**Published:** 2024-12-18

**Authors:** Emma Weschke, Jules Schligler, Isla Hely, Thibaut Roost, Jo‐Ann Schies, Ben Williams, Bartosz Dworzanski, Suzanne C. Mills, Ricardo Beldade, Stephen D. Simpson, Andrew N. Radford

**Affiliations:** ^1^ School of Biological Sciences University of Bristol Bristol UK; ^2^ USR 3278 CRIOBE, BP 1013 PSL Université Paris: EPHE‐UPVD‐CNRS Papetoai, Moorea French Polynesia; ^3^ BBC Studios Bristol UK; ^4^ Andromède Océanologie Mauguio France; ^5^ University College London London UK; ^6^ Faculty of Science University of Bristol Bristol UK; ^7^ Laboratoire d'Excellence ‘CORAIL’ Perpignan France; ^8^ Institut universitaire de France (IUF) Paris France; ^9^ Las Cruces, Pontificia Universidad Católica de Chile Estación Costera de Investigaciones Marinas and Center for Advanced Studies in Ecology and Biodiversity Santiago de Chile Chile

**Keywords:** ALAN, coral reef fish, exposure duration, field experiment, nighttime fish community, nocturnal fish, predators

## Abstract

Artificial light at night (ALAN) is an anthropogenic pollutant that is intensifying and expanding in marine environments, but experimental studies of community‐level effects are generally lacking. The inshore, shallow, and clear‐water locations of coral reefs and their diverse photosensitive inhabitants make these ecosystems highly susceptible to biological disturbances; at the same time, their biodiversity and accessibility make them model systems for wider insight. Here, we experimentally manipulated ALAN using underwater LED lights on a Polynesian reef system to investigate the influence on localised nighttime fish communities compared to control sites without ALAN. We collected infrared video censuses of baseline communities prior to manipulation, which we repeated following short‐term (mean of three nights) and prolonged (mean of 25 nights) exposures to ALAN. Short‐term ALAN exposure did not induce any significant alterations to the nighttime fish community, but prolonged ALAN exposure increased nighttime species richness. Species compositions exposed to prolonged ALAN were more dissimilar from their baseline compared to control sites. The difference between community compositions at prolonged ALAN exposure and control sites was not apparent at the family level; instead, it was observed from the composition of trait guilds. Following prolonged ALAN exposure, more diurnal and nocturnal predatory species (piscivores, invertivores, and planktivores)—particularly those that are site‐attached or mobile within reefs—were present in nighttime assemblages. Our experimental findings show that coastal ALAN could cause trophic imbalances and circadian disturbances in localised nighttime reef fish communities. Given that community‐wide consequences were only apparent after prolonged ALAN exposure suggests that management of the duration of artificial lighting could potentially be used to reduce impacts on marine ecosystems.

## Graphical abstract

Artificial light is a manmade pollutant that is spreading into marine environments, and coral reefs are particularly susceptible to biological impacts due to the inshore, shallow, and clear‐water locations that they thrive in. We manipulated artificial light at night on a Polynesian coral reef to study its impact on nighttime fish communities. An average of three nights exposure to artificial light did not change the composition of fish assemblages; however, an average of twenty‐five nights of artificial light exposure significantly increased fish species richness and the presence of predators at night compared to controls.

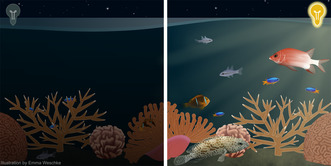

## 1 Introduction

Artificial light at night (ALAN) is a rapidly growing and globally pervasive environmental pollutant, eroding the historic prevalence of naturally dark nights (Cinzano, Falchi, and Elvidge 2001; Hölker et al. 2010). ALAN arises from a multitude of human activities, including transient light from transportation and perpetual light that supports the functioning and safety of residential and industrial areas. In urbanised hotspots (e.g., towns, cities, ports and industrial centres), the scattering of upwardly emitted and reflected artificial light by water molecules and other particles suspended in the atmosphere causes widespread skyglow (Falchi et al. 2016). ALAN interferes with natural cycles of light to which many biological processes are intrinsically tied (Gaston et al. 2017). A burgeoning body of research over the past two decades has documented physiological, reproductive, developmental, behavioural, and navigational impacts on individuals in a diverse range of taxa, as well as population and ecosystem‐wide consequences (Gaston and Bennie 2014; Grubisic et al. 2019; Falcón et al. 2020; Grunst and Grunst 2023). Despite this major progress in understanding the biological consequences of ALAN, investigations of the implications for marine fauna remain scarce, especially at the community level.

Sources of ALAN are predominantly land‐based, so it is unsurprising that terrestrial research has led biological investigations of ALAN. Nonetheless, ALAN spans 22% of coastlines (Davies et al. 2014), reaching depths of 10 m in approximately 1.6 million km^2^ of the world's coastal seas (Smyth et al. 2021). Coral reefs are exposed to ALAN indirectly from the skyglow emitted by coastal residential areas, and directly from transient (e.g., commercial and recreational vessels) and perpetual (e.g., ports, marinas and coastal resorts) sources. It is estimated that 15% of global coral reefs experience brighter nights than natural levels due to anthropogenic activities (Ayalon, Rosenberg, et al. 2021). Many abiotic attributes of the environments where coral reefs thrive, such as shallow depths and clear waters, make reef ecosystems particularly susceptible to ALAN. Scleractinian corals and their algal endosymbionts—foundation organisms that build coral reefs—are highly photosensitive, due to their dependence on natural light cycles for photosynthesis and synchronised spawning (Gorbunov and Falkowski 2002; Kaniewska et al. 2015). Reef fish also rely on natural fluctuations in moonlight for initiating mass spawning migrations (Ikegami et al. 2014), stimulating gonadal development and gamete release (Takemura, Rahman, and Park 2010), enhancing larval growth (Shima and Swearer 2019), and timing of larval settlement (Wantiez, Hebert, and Juncker 2007). Furthermore, light fluctuations influence the periodicity and composition of acoustic choruses produced during peaks in biological activity (McWilliam et al. 2017). Thus, ALAN could cause disruption to many vital biological processes on reefs, yet this pollutant remains one of the most understudied threats to these ecosystems.

Research into the biological effects of ALAN on coral reef organisms has begun to uncover biochemical, physiological and gametogenetic changes in corals (Ayalon, Rosenberg, et al. 2021; Levy et al. 2020). Moreover, impacts on fish larval recruitment, development, reproductive success, nocturnal activity and predator avoidance have been found in aquarium experiments (Fobert, Da Silva, and Swearer 2019; Fobert, Schubert, and Burke Da Silva 2021; O'Connor et al. 2019) and in situ manipulations (Schligler et al. 2021; Georgiou et al. 2023). However, all research on reef fish has focussed on diurnal species, with the direct effects of ALAN on nocturnal species—active during the period of artificial illumination—unstudied. Nocturnal species make up 21%–33% of fish biomass on coral reefs and provide a vital role in cycling nutrients and energy from external nighttime foraging habitats to reefs (Collins, Bellwood, and Morais 2022, 2024; Marnane and Bellwood 2002). Many nocturnal species are also commercially valuable and thus targeted by fisheries. The lack of research into how ALAN affects nocturnal reef species is unsurprising given the logistical challenges in observing nighttime communities in situ, where ecological and behavioural observations need to be conducted without artificial illumination within the visible spectrum of the subject organisms. We are aware of only three studies that have documented community‐scale implications of ALAN on fish in situ, in an estuary (Becker et al. 2013), a river delta (Nelson et al. 2021) and an urban harbour (Bolton et al. 2017). These studies used dual frequency identification SONar (DIDSON) or adaptive resolution imaging sonar (ARIS) to quantify occurrences of fish of different size classes. Whilst this high‐frequency acoustic imaging produces valuable observations, the image resolution of DIDSON and ARIS is too low for species identification, limiting the detail to which community‐level responses can be deduced. Community‐level investigations into the direct effects of ALAN on nocturnally active reef fishes are crucial for a full understanding of the threat posed by this global pollutant to coral reef ecosystem functioning.

Very few studies, in any taxa, have considered how biological responses might change according to the duration of ALAN exposure (Sanders et al. 2021). Where this has been investigated, extended exposure to ALAN elicited stronger biological responses compared with acute exposures; for example, causing further disruption to melatonin production in humans, nocturnal pollination by moths, flight in nocturnally active seabirds, sleep in mice, and sexual signalling in glow worms (Chang et al. 2012; Macgregor et al. 2019; Panagiotou, Rohling, and Deboer 2020; Elgert et al. 2021; Syposz et al. 2021). However, with the exception of Panagiotou, Rohling, and Deboer (2020), who exposed mice to 3 months of ALAN, the maximum exposure duration in these studies ranged from 20 min to one night. Most sources of ALAN in the environment are ever‐present, so it is important to evaluate more environmentally relevant exposure durations. Research in other fields has revealed that animal responses to anthropogenic stressors can change over time: there can be reduced effects through, for example, increased tolerance, desensitisation or habituation, or a greater effect as a result of heightened sensitivity to the stimulus (Bejder et al. 2009). For instance, acute exposure to motorboat noise has been found to elevate ventilation rates in naïve fish, but not in individuals that had been previously exposed to motorboat noise, suggesting that they have habituated to the stressor (Nedelec et al. 2016; Harding et al. 2018). By contrast, cortisol and androgen levels significantly increased in anemonefish exposed to 48 h of motorboat‐noise playback compared to 30 min of exposure, suggesting sensitisation to the stressor (Mills et al. 2020). Scaled‐up, changes in response to prolonged exposure could have community‐wide consequences over time due to the plethora of interacting species that become more vulnerable or tolerant in their altered environment. Advancing our understanding of the relationship between ALAN exposure duration and its effects on wild communities is therefore vital to inform policy solutions that could help to mitigate impacts on the growing extent of artificially illuminated marine habitats.

In this study, we experimentally manipulated ALAN in situ on lagoonal reefs in French Polynesia to assess its effects on the localised nighttime fish community. As coral reefs are highly vulnerable to ALAN, they are a priority marine ecosystem for such a community study. Their renowned productivity and biodiversity also make them an ideal system in which to collect enough survey data from nocturnal species, which are far less numerous than diurnal species. For nighttime surveys, we used an underwater infrared (850 nm) video system—operating outside the visible spectrum for reef fish (Carleton et al. 2020)—as a non‐intrusive nighttime census method that provided sufficient image resolution to score fish occurrences to the genus and, often, the species level. The use of infrared enabled video census under natural dark conditions provided baseline data on nighttime fish communities as a control to compare against those obtained following short‐term and prolonged exposure to ALAN. We predicted that ALAN would significantly alter the local nighttime fish community, both because the greater visual aid for foraging would attract large predatory species (Becker et al. 2013; Dwyer et al. 2013; Manfrin et al. 2017; Brown et al. 2023) and because species lower in the trophic chain would be deterred to minimise their risk of predation (Bengsen et al. 2010; Rotics, Dayan, and Kronfeld‐Schor 2011). We also predicted that ALAN would attract not only nocturnal predators but also diurnal predators due to the potential for light to suppress melatonin production and extend foraging windows (Garber 1978; Brainard et al. 1984; Russ, Rüger, and Klenke 2015; de Jong et al. 2016). Finally, we predicted that prolonged exposures would result in greater changes in the nighttime community compared to short‐term exposures due to the cumulative attraction of predatory species and deterrence of prey species over time.

## 2 Materials and Methods

### 2.1 Experimental Design

Our experiment ran from 10th January to 30th April 2021 across 20 experimental study sites along the lagoonal reefs on the north shore of Mo'orea, French Polynesia (17°28′49.6″ S 149°50′40.0″ W). Sites were selected for the presence of magnificent sea anemones, *Radianthus magnifica* (formerly *Heteractis magnifica* ), inhabited by orange‐fin anemonefish, *Amphiprion chrysopterus* , as they were the focus of parallel ALAN studies. All sites were of comparable reef structure—comprising lagoonal sandy benthos with scattered coral bommies—at depths of 1.1–18.3 m. All sites had very little or no known prior exposure to artificial illumination. We allocated sites pseudo‐randomly to one of two treatments: 10 ‘ALAN’ sites were exposed to ca. 12 h of experimentally generated artificial light after sunset until sunrise each night; and 10 ‘control’ sites experienced natural nighttime light conditions. Sites of the same treatment were a minimum of 10 m apart; sites of different treatments were a minimum of 8 m apart. On a given day, trials were started at two sites, one randomly allocated to each treatment. We conducted community video surveys at three different exposure‐duration timepoints: (1) ‘baseline’, before introducing artificial light in the ALAN treatment; (2) ‘short‐term’, when sites had received 3 ± 1 nights (mean ± SE) of treatment; and (3) ‘prolonged’, when sites had received 25 ± 2 nights of treatment.

We generated ALAN using waterproof LED arrays that were custom built by Dean Chamberlain and Stephen Swearer, University of Melbourne. Each array contained three vertical white‐light LED strips, powered by 12 V lead batteries, emitting 6000–6500 K (peak wavelength of 450–483 nm) to generate 360° of luminance. The white‐light LEDS were used to mimic the most prevalent current sources of light pollution (Gaston, Visser, and Hölker 2015), and were positioned to imitate the point‐source lighting emitted from, for example, marinas, moorings, jetties, and seaside resorts that directly illuminate reefs. A light sensor triggered the LED arrays to turn on after sunset (18:40–17:40 across the duration of the experiment) and off after sunrise (05:34–06:12); the light sensor was not triggered by the LED arrays turning on. Prior to baseline video surveying at a site, we placed a PVC replica of the LED array, that emitted no light, for 30 ± 11 (mean ± SE) nights to acclimate fish to the presence of the artificial structure. The baseline video survey was collected on one night for each site at the end of this period. After baseline surveys were collected, we replaced the PVC replicas with LED arrays at ALAN sites. At control sites, PVC replicas remained and underwent a small physical disturbance to standardise for the interference caused by the switch from replica to LED array at the ALAN sites. We then conducted short‐term and prolonged video surveys (one each per site). For a given site, exposed to either ALAN or control conditions, there were 7 ± 1 nights between baseline and short‐term video surveys and 22 ± 2 nights between short‐term and prolonged video surveys. Consequently, baseline, short‐term and prolonged surveys were scattered across the lunar cycle. Batteries of LED arrays were changed every 3 days and so we applied a similar level of disturbance to PVC replicas at control sites to standardise the interference.

We measured underwater light intensity from an LED array in illuminance (lux; lumens per square metre) using a SpectroSens2+ (Skye Instruments Ltd) light meter, in increments from 1 to 10 m. Lux readings are given at an accuracy typically within 0.008% at 20°C (Table S1; Figure S1). Our study sites were inaccessible at night due to logistical restraints and safety regulations, so illuminance measurements were made on a moonless night (21:44–23:00) at two accessible inshore underwater environments, contrasting in their light propagation potential. One site had white sand benthos with clear water, promoting high illuminance propagation (Ta'ahiamanu, 17°29′29.19″ S 149°51′2.60″ W). The other site had a black sand substrate with turbid water, promoting low light propagation ('Ōpūnohu Bay, 17°31′3.36″ S 149°51′2.07″ W). At the high‐illuminance‐propagation site, light levels were 18.4 ± 3.5 lx (mean ± SE) 1 m from the LED array and had attenuated at 10 m to match natural nighttime light levels on the same night (Table S1a). At the low‐illuminance‐propagation site, light levels were 13.0 ± 2.8 lx 1 m from the LED array and had attenuated at 5 m from the LEDs to match natural levels (Table S1b). Coral structures and bommies were absent at both test sites, yet present at all the study sites. Structured substrate will likely block, reflect, and refract light, so study sites may experience even lower light‐propagation distances than the test sites. Thus, there was no evidence of spillover of experimental light between study sites. Raw lux data are available at Zenodo data repository (Weschke 2024).

### 2.2 Video Surveys

We recorded dusk and nighttime videos at all sites using GoPros (Hero2, Hero3), with lenses that were modified to transmit infrared (IR) wavelengths, paired with waterproof IR lights (850 nm LEDs, custom built by Bartosz Dworzanski, University of Bristol). IR lights were not required to record videos under artificial light at ALAN sites but were nonetheless used to standardise for any possible structural and electronic interference on fish behaviour. We tested the distance at which the GoPros could capture footage detailed enough to identify fish to species level under the control conditions (illuminated only using IR) and ALAN conditions (illuminated with artificial visible light and IR). To do this, we positioned visual markers at increasing 1 m increments from a camera. Under both IR and ALAN lighting conditions, the 1 m marker was clearly visible, the 2 m marker was faintly visible and the 3 m marker was not visible in the video footage.

For each survey, we deployed IR lights and a GoPro camera at the relevant site in late afternoon; lights and camera were turned on at deployment. The IR lights illuminated sites continuously for up to 4 h (limited by battery capacity). GoPros were programmed to record 10‐min videos every 30 min using an intervalometer (CamDo Solutions Time Lapse Intervalometer TL.003AX). Interval recordings ensured collection of the maximum duration of footage as the camera battery allows, as late into the night as possible before the IR light batteries were depleted. From the videos recorded at each site and for each ALAN exposure duration (baseline, short‐term and prolonged), we collected data from one video at dusk and one video at night. Videos recorded closest to the time of sunset were selected for dusk community surveys, with all analysed videos recorded 7.4 ± 0.6 min (mean ± SE) before or after sunset. From nighttime footage, we selected the latest video recording from each night whilst ensuring that all three analysed videos from the same site (one each for the three exposure durations) were recorded within 15 min of each other. All analysed nighttime videos were recorded 74.9 ± 3.3 min after sunset. Videos were available for all three exposure durations at 16 of the 20 sites; due to logistical reasons, we were only able to record during the prolonged exposure duration (not baseline or short‐term exposure) at the remaining four sites.

We surveyed videos that were illuminated solely by IR light (all control videos and baseline videos at the ALAN sites) blind to treatment and exposure duration by assigning them randomly generated coded video labels. The ALAN videos following short‐term and prolonged exposure periods were also surveyed blind to exposure duration using the same unidentifiable‐label approach. For each video, fish species were documented (by E.W.) as present/absent, whether active or resting in the footage. Abundance data could not be collected because it is not possible to distinguish newly arriving from returning individuals in the video footage. Crypto‐benthic fish were excluded from the surveys due to inaccuracy and inconsistency of identification. Uncertainties in fish classification were resolved by Polynesian fish specialist Gilles Siu, CRIOBE. When an individual could not be assigned to a species, they were categorised to the lowest taxonomic level; in rare cases, this was only to the family level (e.g., occasionally for Apogonidae). Survey data are available at Zenodo data repository (Weschke 2024).

### 2.3 Data Analysis

We analysed the α‐diversity (species richness) for dusk and nighttime surveys separately with generalised linear mixed models (GLMMs), using the *lme4* package (Bates et al. 2015) in R studio. Each GLMM had a Poisson distribution and an identity link function (to fit count data without the need for transformation), with treatment (ALAN, control), exposure duration (baseline, short‐term, prolonged), and their interaction included as fixed effects, along with site depth. GLMMs had a matched design to account for the repeated surveys taken from the three exposure durations of each site (*n* = 16 sites), so site was included as a random term. Lunar phase on the night of video survey was also accounted for as a random term containing five levels: new (< 20% illuminated), crescent (20%–40% illuminated), half (> 40%–60% illuminated), gibbous (> 60%–80% illuminated), and full moon (> 80% illuminated). For both species richness models, the fitting of the random effects structure was aided by specifying a weak Bayesian prior using the *bglmer()* function from the *blme* package (Chung et al. 2013). This acts as a wrapper function for lme4 models, fitting a weakly informative prior to the covariance matrix, helping to avoid singular model fit. Neither of the Poisson GLMMs were overdispersed. We generated summary values for fixed terms by comparing a model with and without the relevant term using the *anova()* function in R studio. If the interaction term or the main effect of exposure duration was significant, we used post hoc Tukey's multiple comparisons tests with the package *emmeans* (Lenth 2022) in R studio to explore the effect further. As there was no significant effect of ALAN on fish species richness at dusk (see Section 3), subsequent analyses detailed below were only carried out on nighttime video surveys.

We computed β‐diversity (Sørensen dissimilarity index) to quantify the amount of change in species composition over time, from baseline to short‐term exposure, from baseline to prolonged exposure, and from short‐term to prolonged exposure. Sørensen dissimilarity indices were calculated for each treatment (ALAN, control) using the *beta.temp()* function in the *betapart* package (Baselga and Orme 2012) in R studio, and were then compared using Mann–Whitney *U* tests.

To investigate the effect of prolonged ALAN exposure on the taxonomic and trait composition of nighttime fish communities compared to control conditions, we generated four different datasets. First, we grouped fish species by family. Then, we assigned the fish species across three trait categories (Table S2): trophic guild (piscivores, herbivores/detritivores, invertivores [mobile prey], invertivores [sessile prey], omnivores, and planktivores); temporal niche (diurnal, nocturnal, and cathemeral); and mobility (site‐attached, mobile within a reef, and mobile across a reef). Trait categorisations were based on information from FishBase (Froese and Pauly 2022) and the supplementary material of Mouillot et al. (2013, 2014) and Parravicini et al. (2021). Each of the four datasets contained a matrix of counts (number of species) present from each category (family or trait) per sample (video survey). Comparisons of the composition of families and each of the traits between ALAN and control treatments were conducted using multivariate generalised linear models (GLMs) with the *manyglm()* function in the *mvabund* package (Wang et al. 2012) in R studio. A Poisson distribution family was used in all multivariate GLMs apart from the mobility trait model, which used a negative binomial distribution. Statistical inference was from likelihood ratio tests (LRTs) using the *anova()* function and PIT‐Trap resampling (probability integral transform residual bootstrap) with 9999 iterations. The contribution of taxonomic families and traits towards dissimilarities between communities of each treatment group were identified using percentage similarity analysis (SIMPER; Clarke 1993) via the *simper()* function in the *vegan* package. When analysing taxonomic and trait composition for prolonged exposures, we included the four sites (two of each treatment) for which we had only been able to record video surveys for this exposure period (*n* = 20 sites), while analysis of short‐term exposures remained at (*n* = 16 sites).

To visualise variation in the nighttime composition of ALAN and control communities, we generated separate non‐metric multidimensional scaling (NMDS) ordinations (Clarke and Warwick 2001) for the family, trophic guild, temporal guild and mobility datasets. To do this, we generated Bray‐Curtis resemblance matrices from untransformed counts of species for both short‐term and prolonged exposure durations, using the package *vegan* (Oksanen et al. 2019). Using the *envfit()* function in that package, we assessed variables (families or traits) in each resemblance matrix using permutation for significance in their contribution to the spread of data within the two‐dimensional NMDS space. Only the significant variables (*p* < 0.05) were overlaid as vector loadings onto NMDS plots to indicate the families or traits responsible for driving the greatest variation in communities across sites. The change in the percentage and number of species present from baseline communities to communities exposed to prolonged ALAN or control conditions was calculated for each trait. Data and R code are available at Zenodo data repository (Weschke 2024).

## 3 Results

**FIGURE 1:**
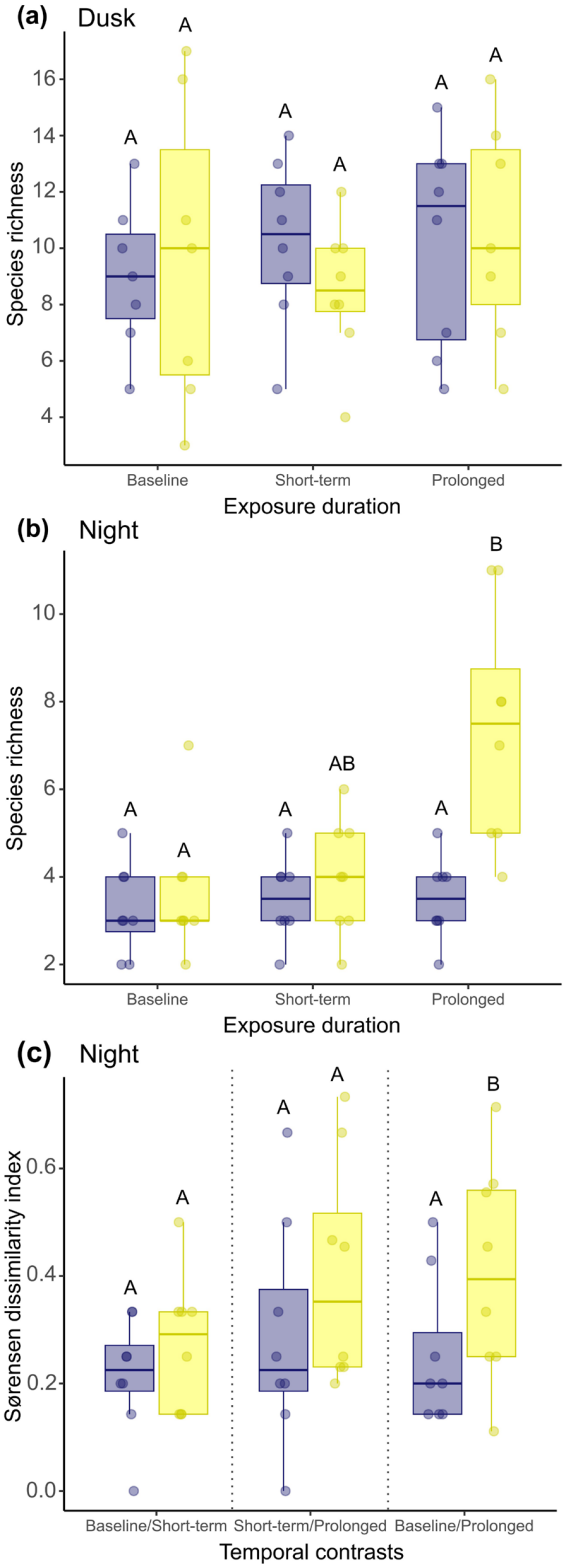
Species richness before (baseline) and after short‐term and prolonged exposure to control conditions and ALAN at (a) dusk and (b) night. (c) Temporal contrasts in the species present (β‐diversity) at night between exposure durations at control and ALAN sites. The plot is split by vertical dashed lines for each temporal contrast test: Pre‐manipulation baseline versus short‐term exposure, short‐term versus prolonged exposure and baseline versus prolonged exposure. β‐diversity index calculated using Sørensen dissimilarity, where a value of 0 indicates all species remain the same and a value of 1 indicates all species are different. In all panels, control sites are represented in blue and ALAN sites are represented in yellow; boxes denote median and interquartile range; whiskers indicate data that fall within 1.5 times the interquartile range; and contrasting letters above bars denote statistical significance. *N* = 16 sites.

Treatment, exposure duration and their interaction had no significant effect on species richness at dusk (Table 1a; Figure 1a). At night, however, there was a significant effect of the interaction between treatment and exposure duration on species richness (Table 1b; Figure 1b). At control sites, there was no significant difference in nighttime species richness across exposure durations (Tukey's multiple comparisons test: all *p* > 0.999). There was also no significant difference in nighttime species richness at ALAN sites between the baseline and short‐term exposure durations (*p* = 0.999). However, after prolonged ALAN exposure, nighttime species richness was 103% higher compared to the baseline level (*p* = 0.021), and there was a non‐significant trend towards greater species richness following prolonged compared to short‐term exposure to ALAN (84% increase, *p* = 0.061). Consequently, nighttime species richness was 111% greater after prolonged ALAN exposure than in equivalent control conditions (*p* = 0.025).

**TABLE 1:** Statistical summary of Poisson GLMMs investigating the effect of light treatment on species richness (a) at dusk and (b) at night. Site and lunar phase were included as random terms (reported in italics). The reference level for treatment is control; the reference level for exposure duration is baseline. Chi‐squared and *p*‐values were obtained from ANOVA model comparisons. Estimate and standard error (SE) values for fixed terms, and variance and SD values for random terms, were obtained from the full GLMM output. Significant effects (*p* < 0.05) are highlighted in bold. *N* = 16 sites in both analyses.

Explanatory variable	Estimate/*variance* ± SE/*SD*	*χ* ^2^	*p*
(a) Species richness—dusk Non‐significant interaction term removed: Treatment × Exposure duration (ANOVA: *χ* ^2^ = 1.35, *df* = 2, *p* = 0.509)			
(Intercept)	9.36 ± 1.34		
Treatment	−0.35 ± 1.34	0.07	0.788
Exposure duration		0.86	0.652
Short‐term	0.30 ± 1.16		
Prolonged	1.10 ± 1.23		
Depth	0.04 ± 0.16	0.05	0.820
Site	*2.68 ± 1.64*		
Lunar phase	*1.61 ± 1.27*		
(b) Species richness—night			
(Intercept)	3.23 ± 0.73		
Treatment	0.27 ± 1.00		
Exposure duration			
Short‐term	0.26 ± 0.93		
Prolonged	0.14 ± 0.98		
Treatment × Exposure duration		**6.50**	**0.039**
Depth	0.02 ± 0.09	0.07	0.796
Site	*0.22 ± 0.47*		
Lunar phase	*0.25 ± 0.50*		

There was no significant difference in β‐diversity between control and ALAN treatments for baseline versus short‐term exposures (Mann–Whitney *U* test: *Z* = −1.17, *n* = 16 *p* = 0.242) and short‐term versus prolonged exposures (*Z* = −1.54, *n* = 16, *p* = 0.123; Figure 1c). However, there was a significant difference in Sørensen pair‐wise dissimilarity between control and ALAN treatments for baseline versus prolonged exposures (*Z* = −2.00, *n* = 16, *p* = 0.045). Species compositions were more dissimilar after prolonged ALAN exposure compared to control conditions (Figure 1c).

ALAN had no significant effect on the taxonomic composition of fish communities at the family level after either short‐term exposure (ManyGLM: LRT = 7.05, *df* = 1, *p* = 0.318; Figure S2a; Table S3a) or prolonged exposure (LRT = 21.89, *df* = 1, *p* = 0.076; Figure S2b; Table S3b). Short‐term ALAN exposure also had no significant effect on the composition of fish from different trophic guilds (LRT = 3.76, *df* = 1, *p* = 0.395), temporal niches (LRT = 1.34, *df* = 1, *p* = 0.448), or mobility types (LRT = 0.61, *df* = 1, *p* = 0.529) in the nighttime community (Figure S3; Table S4). However, prolonged ALAN exposure did affect the composition of trophic guilds, temporal niches, and mobility types in the nighttime fish community (see below).

**FIGURE 2:**
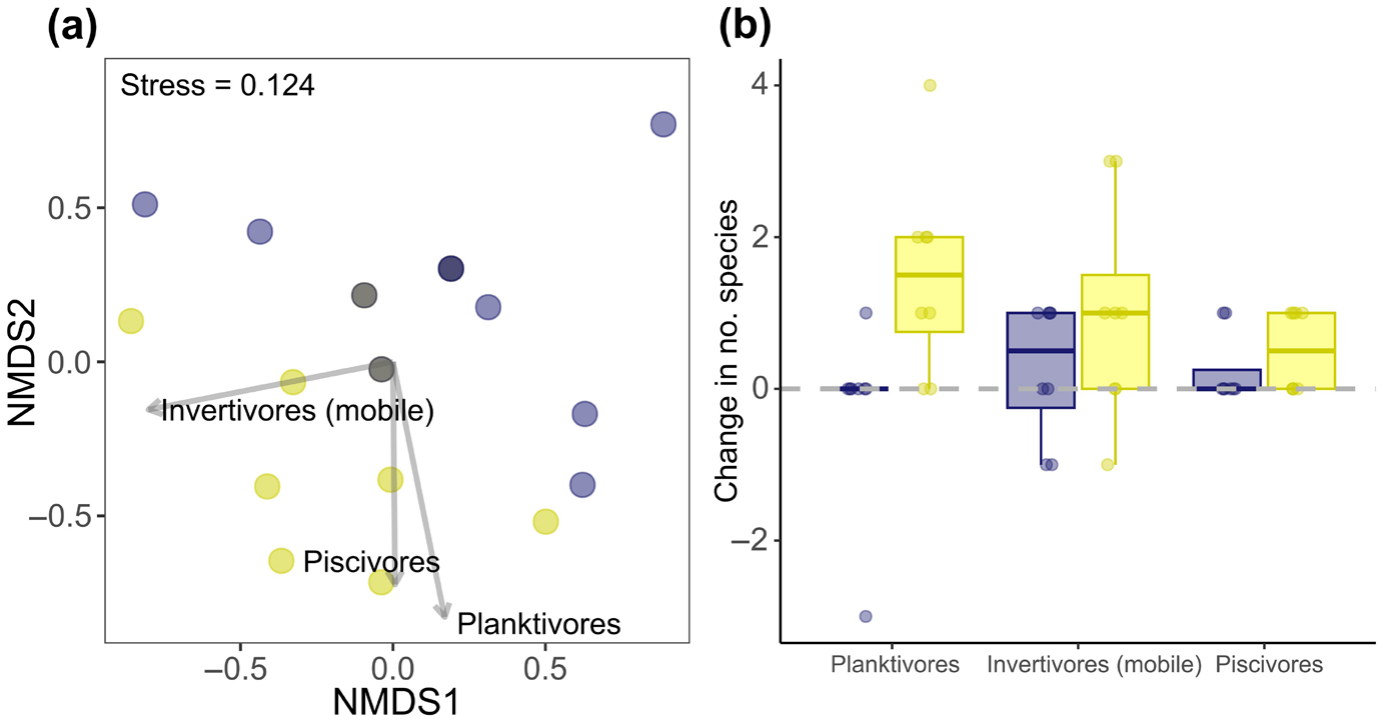
(a) Non‐metric multidimensional scaling (NMDS) ordinations displaying the variation in the composition of trophic guilds in the nighttime fish community. Fitted labelled vectors show the most influential guilds that are driving the spread of data. Data points represent individual sites exposed to prolonged ALAN (yellow) and control conditions (blue); darker points indicate overlapping data of the same treatment while mixed colours indicate overlapping data of different treatments. The stress value obtained indicates good representations of the distribution of trophic guilds. (b) The change (from baseline to prolonged exposure) in the number of species belonging to the three most influential trophic guilds at control (blue) and ALAN (yellow) sites. Boxes denote median and interquartile range; whiskers indicate data that fall within 1.5 times the interquartile range. *N* = 20 sites.

Prolonged ALAN exposure significantly affected the composition of trophic guilds in the nighttime fish community (LRT = 14.0, *df* = 1, *p* = 0.024). SIMPER identified the top three trophic guilds contributing to dissimilarity between prolonged ALAN and control communities (Table 2a) as planktivores (34%), invertivores (mobile prey; 33%) and piscivores (15%), which were the same guilds contributing most to the spread of data within the two‐dimensional NMDS space (Figure 2a). Community assemblages at sites exposed to prolonged control conditions experienced minimal change in trophic guilds compared to baseline (Figure 2b). However, after prolonged exposure to ALAN, there was a 50% ± 25% (mean ± SE; 0.5 ± 0.2 species) increase in piscivorous species, a 94% ± 27% (1.5 ± 0.5 species) increase in planktivorous species and a 96% ± 41% (1.0 ± 0.5 species) increase in invertivorous (mobile prey) species compared to baseline (Figure 2b).

**TABLE 2:** SIMPER analysis output for (a) trophic guild, (b) temporal niche, and (c) mobility type categorisations ordered by greatest contribution toward dissimilarities between nighttime communities exposed to prolonged ALAN versus control conditions.

	Average presence		Contribution %	Cumulative %
	Control	ALAN		
(a) Trophic guild				
Planktivores	1.70	2.80	34	34
Invertivores (mobile)	1.20	2.10	33	67
Piscivores	0.30	0.70	15	82
Omnivores	0.10	0.50	11	93
Herbivores + detritivores	0.00	0.20	4	97
Invertivores (sessile)	0.00	0.20	3	100
(b) Temporal niche				
Diurnal	1.90	3.40	45	45
Nocturnal	1.10	2.20	34	79
Cathemeral	0.30	0.90	21	100
(c) Mobility				
Site attached	2.30	4.10	51	51
Mobile within a reef	1.00	2.20	45	96
Mobile across reefs	0.00	0.20	4	100

**FIGURE 3:**
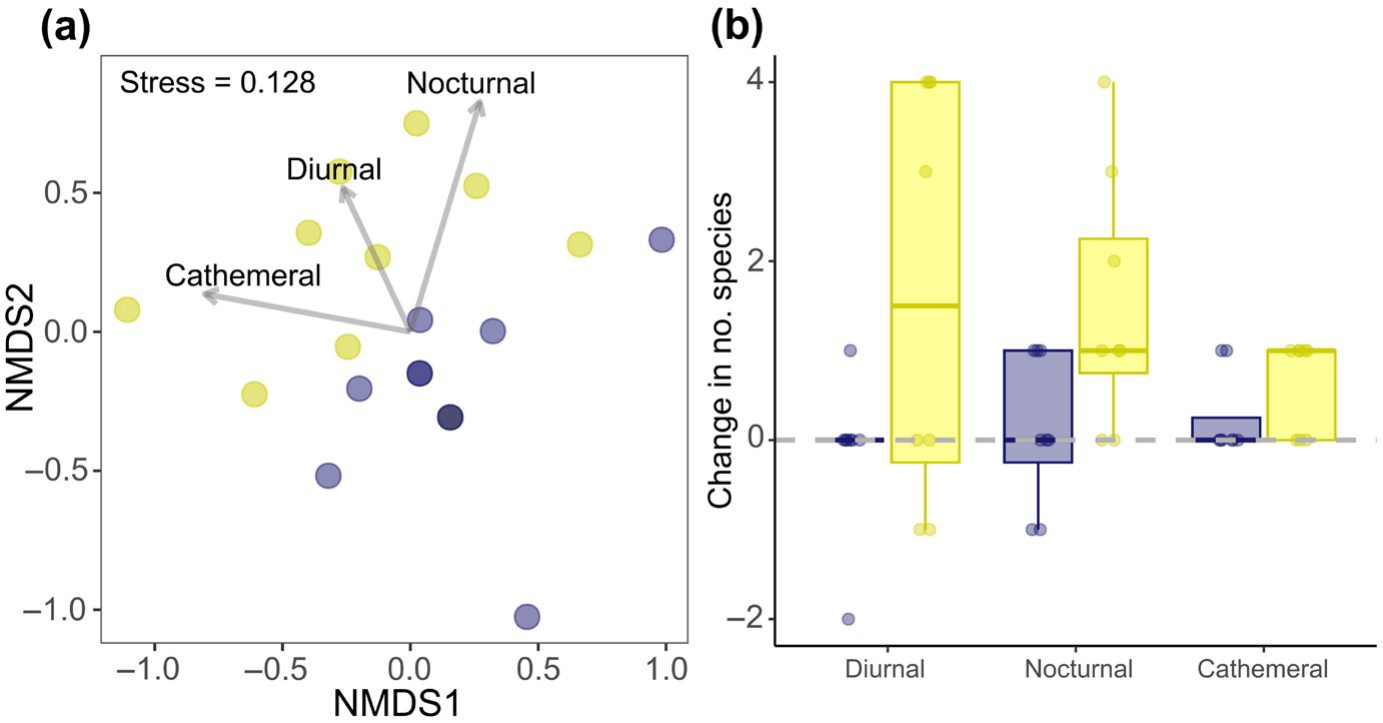
(a) Non‐metric multidimensional scaling (NMDS) ordinations displaying the variation in the composition of temporal niches in the nighttime fish community. Fitted labelled vectors show the most influential niches that are driving the spread of data. Data points represent individual sites exposed to prolonged ALAN (yellow) and control conditions (blue); darker points indicate overlapping data of the same treatment while mixed colours indicate overlapping data of different treatments. The stress value obtained indicates good representations of the distribution of temporal niches. (b) The change (from baseline to prolonged exposure) in the number of species belonging to the three temporal niches at control (blue) and ALAN (yellow) sites. Boxes denote median and interquartile range; whiskers indicate data that fall within 1.5 times the interquartile range. *N* = 20 sites.

Prolonged ALAN exposure also had a significant effect on the composition of temporal niches present in nighttime fish communities (LRT = 11.18, *df* = 1, *p* = 0.022). SIMPER identified that all three temporal niches characterised in this study contributed strongly to the dissimilarity between prolonged ALAN and control communities (Table 2b): diurnal (45%), nocturnal (34%) and cathemeral (21%). At control sites, community assemblages showed minimal change in the presence of temporal niches from the baseline to the prolonged‐exposure period (Figure 3b). However, after prolonged ALAN exposure, there was a 100% ± 10% (mean ± SE; 0.6 ± 0.2 species) increase in cathemeral species, a 103% ± 48% (1.6 ± 0.8 species) increase in diurnal species and a 142% ± 89% (1.5 ± 0.5 species) increase in nocturnal species compared to baseline (Figure 3b).

**FIGURE 4:**
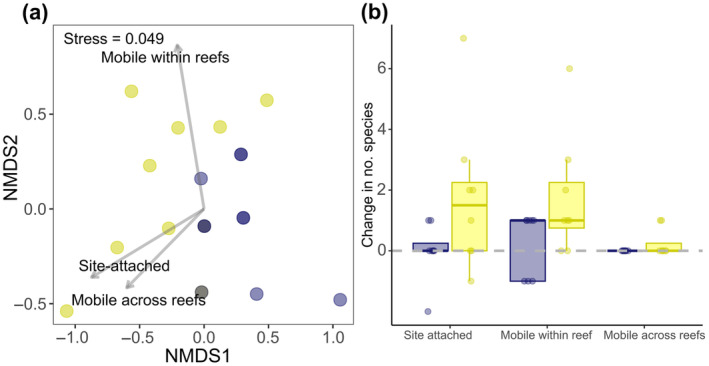
(a) Non‐metric multidimensional scaling (NMDS) ordinations displaying the variation in the composition of mobility types in the nighttime fish community. Fitted labelled vectors show the most influential types that are driving the spread of data. Data points represent individual sites exposed to prolonged ALAN (yellow) and control conditions (blue); darker points indicate overlapping data of the same treatment while mixed colours indicate overlapping data of different treatments. The stress value obtained indicates good representations of the distribution of mobility types. (b) The change (from baseline to prolonged exposure) in the number of species belonging to the three mobility types at control (blue) and ALAN (yellow) sites. Boxes denote median and interquartile range; whiskers indicate data that fall within 1.5 times the interquartile range. *N* = 20 sites.

Finally, prolonged ALAN exposure had a significant effect on the composition of mobility types present in the nighttime fish community (LRT = 10.84, *df* = 1, *p* = 0.031). SIMPER identified that site‐attached species (51%) and those that are mobile within a reef (45%) contributed most to the dissimilarity between prolonged ALAN and control communities, with species that are mobile across reefs (4%) of comparatively little influential (Table 2c). Community assemblages exposed to prolonged control conditions experienced minimal change in the presence of mobility types compared to baseline (Figure 4b). However, prolonged ALAN exposure led to an 81 ± 34% (mean ± SE; 1.8 ± 0.9 species) increase in site‐attached species and a 230% ± 84% (1.8 ± 0.7 species) increase in species that are mobile within a reef compared to baseline (Figure 4b).

## 4 Discussion

To our knowledge, this is the first experimental study to demonstrate that ALAN can alter the nighttime fish community in the marine environment. Short‐term exposure to ALAN (mean duration of 3 nights) on a Polynesian coral reef had little detectable effect on the nighttime fish community. By contrast, prolonged ALAN exposure (mean duration of 25 nights) caused localised increases in species richness compared to the baseline and control reefs at the same time point. ALAN attracted nocturnal and diurnal predatory fishes (secondary consumers and higher), that are site‐attached and mobile within a reef, suggesting that this anthropogenic disturbance could drive localised changes in trophic dynamics, circadian rhythms, the dispersal of populations and thus ecosystem functioning. We used this coral reef as a model system but the ecologically relevant findings from our field‐based manipulation are likely widely relevant and can inform predictions of how other aquatic ecosystems might fare as light pollutant grows in both scale and intensity.

The increased species richness that we found in nighttime surveys following prolonged ALAN exposure could be explained in several ways. It could simply be the consequence of improved visibility to the observer during community census. However, the IR set‐up was the same for all sites, and the visibility range in the footage was equivalent between ALAN and control sites. In principle, increased species richness could result from elevated recruitment of site‐attached species given the positive phototaxis exhibited by many settlement‐stage larval fish (Doherty 1987; Mueller and Neuhauss 2010). But, as the prolonged ALAN manipulation lasted approximately a month, any recruits that had settled within that window would be difficult to detect, let alone classify, in the infrared footage. As such, variation in larval settlement is unlikely to explain the elevated species richness identified in this study. Another potential explanation could be seasonal shifts in communities. However, the dissimilarity in the community composition between baseline and prolonged exposures was significantly greater for the ALAN treatment compared to controls that were monitored at the same time, suggesting that the alteration was driven by prolonged artificial light rather than seasonal shifts. It is therefore likely that the increased species richness under prolonged ALAN is driven by the attraction of transient foraging species responding to elevated prey availability and visual foraging cues in such areas. If mobile species are migrating into more illuminated habitat, there is likely a decrease in species in neighbouring dark environments. Such alterations, and resulting patchy distributions, are likely to have consequences for many interactions between both conspecifics and heterospecifics, including competition for territories, symbiotic relationships, predator–prey dynamics, and the distribution of resources and movement of nutrients through the environment. As an ALAN‐related increase in species richness has also been identified in previous studies of intertidal sandy shores and forest arthropods (Brown et al. 2023; Garratt, Jenkins, and Davies 2019), we might expect this to be a common trend in communities across different habitats.

We did not uncover any major alterations to the taxonomic composition of the nighttime fish community at the family level in response to prolonged ALAN. But when assessing changes to the trophic structure, we found that prolonged ALAN caused increases in piscivorous and planktivorous species, as well as those that feed on mobile invertebrates, compared to baseline nighttime communities. Consequentially, the prevalence of predators on artificially lit reefs was substantially greater, aligning with our predictions and findings across other aquatic and terrestrial ecosystems (Davies, Bennie, and Gaston 2012; Becker et al. 2013; Dwyer et al. 2013; Manfrin et al. 2017; Brown et al. 2023). Species that were observed the most at sites exposed to prolonged ALAN, whilst absent from the majority of prolonged control surveys, included a diurnal planktivore ( *Dascyllus flavicaudus* ), a nocturnal planktivore (*Ostorhinchus nigrofasciatus*), and a cathemeral invertivore ( *Lutjanus fulvus* ), and piscivore ( *Epinephelus merra* ). Many terrestrial and aquatic invertebrates are positively phototactic for orientational and navigational purposes (Dojmi Di Delupis and Rotondo 1988; Shimoda and Honda 2013) and artificial light on coral reefs has been found to attract higher abundances of amphipod species—dominant members of emergent zooplankton and an important prey item for nocturnal fishes (DeFelice and Parrish 2003)—compared to dark ambient conditions (Navarro‐Barranco and Hughes 2015). From video observation alone, it was evident that LED arrays were swarmed by small invertebrates that were likely also members of the emergent demersal zooplankton. Many settlement‐stage larval reef fishes also display positive phototaxis at night, which has led to the development of light trapping as an effective method for larval fish collection (Doherty 1987; Mueller and Neuhauss 2010). The concentrated density of illuminated and easily attainable prey is likely a strong factor driving increases in predator prevalence (Perkin et al. 2011), as active feeding behaviours were often displayed by fishes in videos from sites exposed to ALAN (pers. obs.). Elevated localised nighttime predation under lit conditions in marine environments could have severe consequences for zooplankton, invertebrates and fishes. These organisms have evolved to take advantage of reduced predation risk from visual hunters on naturally dark nights to perform essential yet vulnerable behaviours such as feeding, spawning and settlement. Patches of marine habitat that receive chronic ALAN exposure may eventually deplete in lower trophic groups due to the heightened predation risk, with knock‐on consequences for other members of the community.

Though there were substantial increases in the number of piscivorous, planktivorous, and invertivorous fish following prolonged ALAN exposure, there was no equivalent change in the occurrence of omnivores, herbivores, and detritivores. In a previous aquarium experiment, ALAN also had no significant effect on the grazing behaviours of marine urchins and snails (Bauer et al. 2022). This lack of an impact on omnivores, herbivores, and detritivores may be because their food supply is far less likely to be affected in a meaningful way by ALAN. For instance, algae and detritus do not display rapid positive phototaxis. Moreover, whilst ALAN has been shown to induce a small boost in photosynthesis and abundance of both phytoplankton in the water column and algal symbionts in corals (Ayalon, Benichou, et al. 2021; Diamantopoulou et al. 2021), the resulting increase is likely negligible in comparison to the difference between sunny and overcast days. To date, no study has investigated the effect of ALAN on the growth rates of marine benthic microalgae, filamentous algae, and macroalgae—the dominant food of herbivorous reef fishes—but, in a freshwater tank experiment, algal biomass did not change after a 2‐week exposure to ALAN (Czarnecka et al. 2019). Even if ALAN was to cause an increase in growth or abundance of benthic micro‐ and macroalgae on coral reefs, that greater food source would be apparent the next day because it is attached to the substrate. As such, herbivores should not need to forage outside their natural daytime temporal niche, when they are safer from predators, to capitalise on any foraging benefits resulting from ALAN. This contrasts the situation for piscivores, planktivores and invertivores, who would need to increase nighttime activity to profit from ALAN‐induced increases in their prey of fish, phototaxic zooplankton, and mobile invertebrates that will retreat to shelter during the day.

The number of predatory species across all temporal niches (nocturnal, diurnal, and cathemeral) detected in nighttime communities increased from baseline to prolonged ALAN exposures. This increase aligns with our predictions and outcomes from other aquatic and terrestrial ecosystems (Becker et al. 2013; Bolton et al. 2017; Manfrin et al. 2017). Diurnal predators likely remained active beyond their usual daytime foraging window to take advantage of the extended, increased and concentrated availability of prey items (Baxter‐Gilbert et al. 2021), exacerbated by the potential for ALAN to disturb physiological processes involved in circadian regulation, such as suppressing melatonin production (Grubisic et al. 2019). When considering mobility types, we found that prolonged ALAN caused increases in site‐attached species and those that are mobile within a reef. Many of the diurnal site‐attached species (e.g., *D. flavicaudus* and *Pomacentrus coelestis* ) observed following prolonged ALAN exposure would have likely been present under natural dark conditions but remained hidden from video observation, resting within their coral/substrate refugia. It is therefore important to consider the increase in presence of site‐attached diurnal species as a reflection of a change in their activity pattern, and thus visibility to the observer, rather than the influence of ALAN on their distribution across reefs, at least for the exposure duration in this study. However, there may also be differences in occurrence. Though the navigation mechanisms and passage of nocturnal fishes within a reef system at night remain largely unknown, attraction toward light may lure them off course, with repeated nights of illumination establishing and reinforcing a preferred route in their patrol for prey. Scaled‐up over time, this might result in a cumulative effect where more nocturnal predators that are mobile within a reef and even across reefs would chance upon the illuminated area. A longer exposure duration spanning reproductive and recruitment seasons would allow for an assessment into multi‐generational effects on the distribution of site‐attached diurnal species. If ALAN changes the temporal activity patterns of species, it likely creates novel interactions between naturally nocturnal species and unnaturally active diurnal species that would otherwise be resting. The resulting novel nighttime community with traditionally nocturnal and diurnal species simultaneously active has been termed the ‘night light niche’ (Fobert et al. 2023; Garber 1978). It remains unknown whether the night light niche identified in our study represents the community reaching a new, equilibrated, enduring state or whether it is a temporary intermediate phase while the most‐benefiting species gradually begin to dominate at the expense of those who are less successful in more‐illuminated conditions. Field‐based community surveys on reefs exposed to permanent fixtures of light pollution will prove valuable in investigating the final ecological state of the night light niche under chronic ALAN.

In comparison to prolonged ALAN exposure, short‐term exposure did not elicit significant community‐scale changes at any level across taxonomic or trait guilds in our experiment. There are no clear indicators from our short‐term exposure surveys whether species with particular traits were drawn to ALAN sooner than others; the patterns seen after prolonged exposure were not obviously apparent earlier on. As with any predatory attractant, such as baiting and light fishing, there is likely a delay after the initial release of the attractant before a predatory response; often that response intensifies and becomes more rapid when repeated in the same area over time (Clarke et al. 2022; Laroche et al. 2007). It is also possible that there are some finer‐scale impacts of short‐term ALAN exposure on physiology, rest–wake rhythms and behaviours in site‐attached species inhabiting the lit region, but such effects would not be detected using community‐census methods. The minimal effect of short‐term ALAN compared to prolonged exposure in our study suggests that transient light sources, or implementation of intermittent dark periods, especially during known spawning and settlement periods, could cause less ecological disturbance than permanent coastal light fixtures. Motion‐activated street‐lighting with timers is a new measure that has been implemented in the Sunshine Coast, Queensland, to minimise artificial light that can fatally disorient loggerhead turtle ( *Caretta caretta* ) hatchlings. Our experimental white LED lights imitate isolated point sources that reef fish would experience directly from urban structures, such as seaside resorts, harbour walls, and ports, as well as moored vessels. Many of these light sources and their illumination intensities exceed functional needs or exist only for aesthetics, and often their implementation has not considered the innate ability of humans to adjust ocularly in low light. Moreover, artificial light consumes 19% of global electricity production, accounting for greenhouse gas emissions of 1900 Mt. of CO_2_ per year (Hölker et al. 2010). Minimising unnecessary light emissions by intensity, biologically impactful wavelengths and duration of use would be a quick, simple, energy‐efficient, cost‐effective, and climate‐friendly strategy that can be implemented to provide immediate benefit to wildlife (Jägerbrand and Bouroussis 2021).

Whilst ALAN transmission into marine environments is substantial and increasing, and a growing body of research is revealing the negative impacts on biological systems, there remains a significant knowledge gap about the ecological implications of ALAN on marine communities (Marangoni et al. 2022). Our study aimed to provide initial insight into the impact on nighttime communities by using a tropical coral reef as a model system. Coral reefs are a priority ecosystem for this area of research due to the abiotic attributes of the environment in which they thrive, the highly photosensitive organisms that inhabit them, and their proximity to human activity. But they are also home to a high density and biodiversity of nocturnally active species, which makes them ideal for the sampling of sufficient survey data to allow for thorough statistical analysis and meaningful conclusions. Our experimental results provide evidence that the addition of ALAN to a coral reef substantially alters the composition of localised nighttime fish communities by attracting and/or promoting activity in nocturnal and diurnal predators that are both site‐attached and mobile within the reef. This likely has knock‐on effects for the physiology and fitness of diurnal species foraging beyond their natural window of activity, causing trophic imbalances, creating novel interactions, and generating competition among species occupying the unnatural night light niche. Ultimately, there may be changes in the dispersal and movement of resources and nutrients through the reef ecosystem. Research into the ecological implications for reefs and other marine environments that have been exposed to permanently established ALAN would progress our understanding of how this pollutant shapes ecosystems over generations. Such sites would also provide viable study systems for trialling the effectiveness of mitigation measures to limit the transmission of ALAN into the marine environment and thus its impact on vulnerable marine life.

## Author Contributions

**Emma Weschke:** conceptualization, formal analysis, funding acquisition, investigation, methodology, resources, writing – original draft, writing – review and editing. **Jules Schligler:** conceptualization, investigation, methodology, writing – review and editing. **Isla Hely:** investigation. **Thibaut Roost:** investigation. **Jo‐Ann Schies:** investigation. **Ben Williams:** investigation. **Bartosz Dworzanski:** resources. **Suzanne C. Mills:** conceptualization, funding acquisition, investigation, methodology, resources, supervision, writing – review and editing. **Ricardo Beldade:** investigation, methodology, supervision, writing – review and editing. **Stephen D. Simpson:** conceptualization, investigation, supervision, writing – review and editing. **Andrew N. Radford:** conceptualization, investigation, supervision, writing – original draft, writing – review and editing.

## Conflicts of Interest

The authors declare no conflicts of interest.

## Supporting information


Data S1.

## Data Availability

The data that support the findings of this study are openly available in a Zenodo data repository at http://doi.org/10.5281/zenodo.14266460. Due to large file sizes sample survey videos are provided and further videos are available upon request.
