# Supplementary material for: Artificial Light Increases Nighttime Prevalence of Predatory Fishes, Altering Community Composition on Coral Reefs
Source: Glob Chang Biol. 2024 Dec 18;30(12):e70002. doi: 10.1111/gcb.70002 (PMC11653166; doi:10.1111/gcb.70002)
Supplement: Supplementary file 1 — Data S1. [file GCB-30-e70002-s001.docx]

**Supplementary Material**

**Artificial light increases nighttime prevalence of predatory fishes,
altering community composition on coral reefs**

**Emma Weschke, Jules Schligler, Isla Hely, Thibaut Roost, Jo-Ann Schies, Ben Williams, Bartosz Dworzanski, Suzanne C. Mills, Ricardo Beldade, Stephen D. Simpson & Andrew N. Radford**

Additional figures and tables, provided in the order in which they are first referred to in the main paper.


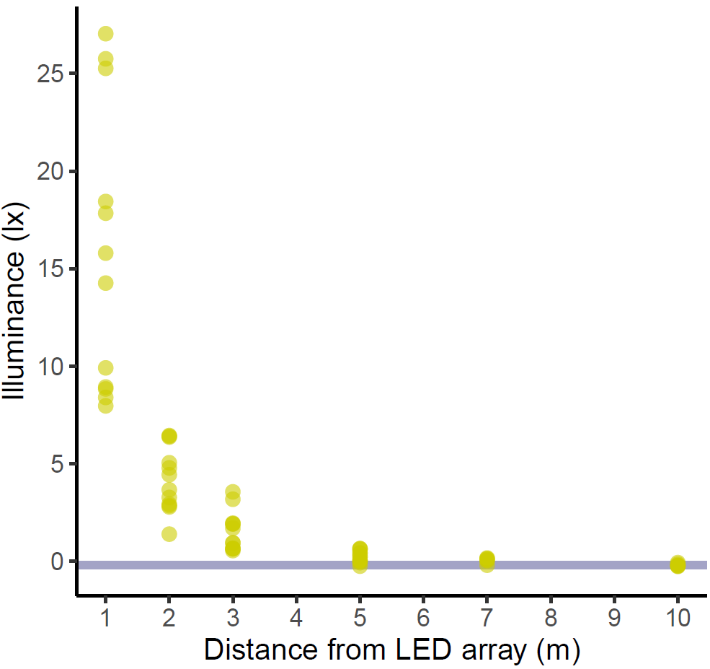


**Figure S1.** Underwater illuminance measurements (lux), taken during a moonless night using a SpectroSens2+ (Skye Instruments Ltd) light meter, at increasing distances from the source of ALAN manipulation (underwater LED arrays). Lux readings are given at an accuracy typically within 0.008% at 20°C. Datapoints are the lux measurements recorded using two types of LEDs (old and new), at both high-illuminance-propagation and low-illuminance-propagation test sites (see Supplementary Table S1). Blue reference band denotes the range of underwater lux measurements under ambient nighttime natural light.

**Table S1.** Illuminance measurements (lux), taken during a moonless night using a SpectroSens2+ (Skye Instruments Ltd) light meter, at increasing distances from old and new underwater LED arrays used to manipulate ALAN in the study. Lux was recorded across two sites contrasting in their propagation potential: a) Ta'ahiamanu – white sand and oligotrophic water; and b) 'Ōpūnohu Bay – black sand and oligotrophic water. LEDs were positioned underwater at depths of 20–30 cm. Lux readings are given at an accuracy typically within 0.008% at 20°C.

| a) Ta’ahiamanu – *high illuminance propagation* | | |  | b) 'Ōpūnohu Bay – *low illuminance propagation* | | | | | |
| --- | --- | --- | --- | --- | --- | --- | --- | --- | --- |
| **Control** | | |  | **Control** | | | | | |
| *Repeat* | *Illuminance (lux)* | |  | *Repeat* | | *Illuminance (lux)* | | | |
| 1 | -0.39 | |  | 1 | | -0.14 | | | |
| 2 | -0.14 | |  | 2 | | -0.13 | | | |
| 3 | -0.16 | |  | 3 | | -0.18 | | | |
| **ALAN Treatment** | | |  | **ALAN Treatment** | | | | | |
| *Distance* | *Illuminance (lux)* | |  | *Distance* | *Illuminance (lux)* | | | | |
| *(m)* | *New LEDs* | *Old LEDs* |  | *(m)* | | *New LEDs* | | *Old LEDs* | |
| 1 | 25.27 | 14.26 |  | 1 | | 18.45 | | 8.84 | |
| 1 | 25.76 | 8.40 |  | 1 | | 17.85 | | 8.94 | |
| 1 | 27.04 | 9.92 |  | 1 | | 15.80 | | 7.97 | |
| 2 | 6.42 | 4.79 |  | 2 | | 1.40 | | 2.86 | |
| 2 | 6.37 | 5.06 |  | 2 | | 3.28 | | 2.79 | |
| 2 | 6.45 | 4.44 |  | 2 | | 3.67 | | 2.94 | |
| 3 | 3.57 | 1.96 |  | 3 | | 0.95 | | 0.65 | |
| 3 | 1.93 | 1.70 |  | 3 | | 0.95 | | 0.68 | |
| 3 | 3.19 | 1.92 |  | 3 | | 0.65 | | 0.56 | |
| 5 | 0.65 | 0.24 |  | 5 | | -0.24 | | 0.24 | |
| 5 | 0.62 | 0.43 |  | 5 | | -0.01 | | 0.03 | |
| 5 | 0.66 | 0.42 |  | 5 | | -0.04 | | -0.06 | |
| 7 | 0.14 | -0.19 |  |  | | |  | |  |
| 7 | 0.18 | -0.03 |  |  | | |  | |  |
| 7 | 0.12 | -0.07 |  |  | | |  | |  |
| 10 | -0.14 | -0.23 |  |  | | |  | |  |
| 10 | -0.06 | -0.20 |  |  | | |  | |  |
| 10 | -0.26 | -0.22 |  |  | | |  | |  |

**Table S2.** Fish traits assigned to surveyed species. Trait classifications were based on information from FishBase (Froese and Pauly, 2022) and published literature (Mouillot et al., 2013, 2014; Parravicini et al., 2021). Herbivore/detritivore = feeds on turf or filamentous algae and/or undefined organic material; invertivore (mobile) = targets mobile invertebrates (i.e., crustaceans and gastropods); invertivore (sessile) = targets sessile invertebrates (i.e., corals, sponges and ascidians); omnivore = feed on both vegetal and animal matter; piscivore = feed on fish and cephalopods; planktivore = feed on zooplankton or phytoplankton (Parravicini et al., 2021). Diurnal = active during the day; nocturnal = active at night; cathemeral = can be active during the day and at night. Site attached = a restricted home range from less than 1 m^2^ up to a few 100 m^2^; mobile within reef = a restricted but large home range from 100 m^2^ to several hectares within a reef; mobile across reefs = will circulate over a large extent of reefs and may change from one reef to another within a short period of time (Mouillot et al., 2013).

| **Species** | **Family** | **Trophic guild** | **Diel activity pattern** | **Mobility** |
| --- | --- | --- | --- | --- |
| *Acanthurus nigricans* | Acanthuridae | Herbivore/detritivore | Diurnal | Site attached |
| *Acanthurus nigricauda* | Acanthuridae | Herbivore/detritivore | Diurnal | Mobile within reef |
| *Acanthurus nigrofuscus* | Acanthuridae | Herbivore/detritivore | Diurnal | Site attached |
| *Acanthurus pyroferus* | Acanthuridae | Herbivore/detritivore | Diurnal | Site attached |
| *Acanthurus triostegus* | Acanthuridae | Herbivore/detritivore | Diurnal | Mobile across reefs |
| *Acanthurus xanthopterus* | Acanthuridae | Herbivore/detritivore | Diurnal | Mobile within reef |
| *Ctenochaetus striatus* | Acanthuridae | Omnivore | Diurnal | Site attached |
| *Zanclus cornutus* | Acanthuridae | Omnivore | Diurnal | Mobile within reef |
| *Zebrasoma scopas* | Acanthuridae | Herbivore/detritivore | Diurnal | Mobile within reef |
| *Cheilodipterus macrodon* | Apogonidae | Piscivore | Nocturnal | Site attached |
| *Cheilodipterus quinquelineatus* | Apogonidae | Piscivore | Nocturnal | Site attached |
| *Ostorhinchus angustatus* | Apogonidae | Invertivore (mobile) | Nocturnal | Site attached |
| *Ostorhinchus apogonoides* | Apogonidae | Invertivore (mobile) | Nocturnal | Site attached |
| *Ostorhinchus nigrofasciatus* | Apogonidae | Planktivore | Nocturnal | Site attached |
| *Ostorhinchus novemfasciatus* | Apogonidae | Planktivore | Nocturnal | Site attached |
| *Pristiapogon exostigma* | Apogonidae | Invertivore (mobile) | Nocturnal | Site attached |
| *Pristiapogon fraenatus* | Apogonidae | Invertivore (mobile) | Nocturnal | Site attached |
| *Pristiapogon kallopterus* | Apogonidae | Planktivore | Nocturnal | Site attached |
| *Balistapus undulatus* | Balistidae | Invertivore (mobile) | Diurnal | Mobile within reef |
| *Melichthys vidua* | Balistidae | Planktivore | Diurnal | Mobile across reefs |
| *Pseudobalistes flavimarginatus* | Balistidae | Invertivore (mobile) | Diurnal | Mobile within reef |
| *Rhinecanthus aculeatus* | Balistidae | Invertivore (mobile) | Diurnal | Site attached |
| *Sufflamen bursa* | Balistidae | Invertivore (mobile) | Diurnal | Site attached |
| *Platybelone argalus* | Belonidae | Piscivore | Cathemeral | Mobile across reefs |
| *Aspidontus dussumieri* | Blenniidae | Omnivore | Diurnal | Site attached |
| *Caranx melampygus* | Carangidae | Piscivore | Cathemeral | Mobile across reefs |
| *Caranx papuensis* | Carangidae | Piscivore | Cathemeral | Mobile across reefs |
| *Selar crumenophthalmus* | Carangidae | Planktivore | Nocturnal | Mobile across reefs |
| *Carcharhinus melanopterus* | Carcharhinidae | Piscivore | Cathemeral | Mobile across reefs |
| *Chaetodon auriga* | Chaetodontidae | Invertivore (mobile) | Diurnal | Site attached |
| *Chaetodon citrinellus* | Chaetodontidae | Invertivore (sessile) | Diurnal | Site attached |
| *Chaetodon lunula* | Chaetodontidae | Invertivore (sessile) | Cathemeral | Site attached |
| *Chaetodon lunulatus* | Chaetodontidae | Invertivore (sessile) | Diurnal | Site attached |
| *Chaetodon ornatissimus* | Chaetodontidae | Invertivore (sessile) | Diurnal | Site attached |
| *Chaetodon quadrimaculatus* | Chaetodontidae | Invertivore (sessile) | Diurnal | Site attached |
| *Chaetodon reticulatus* | Chaetodontidae | Invertivore (sessile) | Diurnal | Site attached |
| *Chaetodon ulietensis* | Chaetodontidae | Omnivore | Diurnal | Site attached |
| *Chaetodon vagabundus* | Chaetodontidae | Invertivore (mobile) | Diurnal | Site attached |
| *Heniochus chrysostomus* | Chaetodontidae | Invertivore (mobile) | Diurnal | Site attached |
| *Himantura fai* | Dasyatidae | Piscivore | Diurnal | Mobile across reefs |
| *Diodon hystrix* | Diodontidae | Invertivore (mobile) | Cathemeral | Mobile within reef |
| *Myripristis berndti* | Holocentridae | Planktivore | Nocturnal | Mobile within reef |
| *Myripristis kuntee* | Holocentridae | Planktivore | Nocturnal | Mobile within reef |
| *Myripristis pralinia* | Holocentridae | Planktivore | Nocturnal | Mobile within reef |
| *Myripristis violacea* | Holocentridae | Planktivore | Nocturnal | Mobile within reef |
| *Myripristis woodsi* | Holocentridae | Planktivore | Nocturnal | Mobile within reef |
| *Neoniphon sammara* | Holocentridae | Planktivore | Nocturnal | Mobile within reef |
| *Sargocentron caudimaculatum* | Holocentridae | Invertivore (mobile) | Nocturnal | Mobile within reef |
| *Sargocentron diadema* | Holocentridae | Invertivore (mobile) | Nocturnal | Mobile within reef |
| *Sargocentron microstoma* | Holocentridae | Invertivore (mobile) | Nocturnal | Mobile within reef |
| *Sargocentron tiere* | Holocentridae | Invertivore (mobile) | Nocturnal | Mobile within reef |
| *Sargocentron tieroides* | Holocentridae | Invertivore (mobile) | Nocturnal | Mobile within reef |
| *Cheilinus chlorourus* | Labridae | Invertivore (mobile) | Diurnal | Mobile within reef |
| *Cheilinus trilobatus* | Labridae | Invertivore (mobile) | Diurnal | Mobile within reef |
| *Epibulus insidiator* | Labridae | Piscivore | Diurnal | Mobile within reef |
| *Gomphosus varius* | Labridae | Invertivore (mobile) | Diurnal | Mobile within reef |
| *Halichores hortulanus* | Labridae | Invertivore (mobile) | Diurnal | Mobile within reef |
| *Halichores trimaculatus* | Labridae | Invertivore (mobile) | Diurnal | Mobile within reef |
| *Labroides dimidiatus* | Labridae | Invertivore (mobile) | Diurnal | Site attached |
| *Pseudocheilinus hexataenia* | Labridae | Invertivore (mobile) | Diurnal | Site attached |
| *Thalassoma hardwicke* | Labridae | Invertivore (mobile) | Diurnal | Mobile within reef |
| *Gnathodentex aureolineatus* | Lethrinidae | Invertivore (mobile) | Nocturnal | Mobile within reef |
| *Monotaxis grandoculis* | Lethrinidae | Invertivore (mobile) | Nocturnal | Mobile within reef |
| *Lutjanus bohar* | Lutjanidae | Piscivore | Nocturnal | Mobile across reefs |
| *Lutjanus fulvus* | Lutjanidae | Invertivore (mobile) | Cathemeral | Mobile within reef |
| *Mulloidichthys flavolineatus* | Mullidae | Invertivore (mobile) | Diurnal | Mobile within reef |
| *Mulloidichthys vanicolensis* | Mullidae | Invertivore (mobile) | Diurnal | Mobile within reef |
| *Parupeneus barberinus* | Mullidae | Invertivore (mobile) | Diurnal | Mobile within reef |
| *Parupeneus multifasciatus* | Mullidae | Invertivore (mobile) | Diurnal | Mobile within reef |
| *Echidna nebulosa* | Muraenidae | Invertivore (mobile) | Nocturnal | Site attached |
| *Gymnothorax javanicus* | Muraenidae | Piscivore | Nocturnal | Site attached |
| *Ostracion meleagris* | Ostraciidae | Invertivore (sessile) | Diurnal | Mobile within reef |
| *Centropyge bispinosa* | Pomacanthidae | Herbivore/detritivore | Diurnal | Site attached |
| *Centropyge flavissima* | Pomacanthidae | Herbivore/detritivore | Diurnal | Site attached |
| *Abudefduf septemfasciatus* | Pomacentridae | Omnivore | Diurnal | Site attached |
| *Abudefduf sexfasciatus* | Pomacentridae | Planktivore | Diurnal | Site attached |
| *Abudefduf sordidus* | Pomacentridae | Omnivore | Diurnal | Site attached |
| *Amphiprion chrysopterus* | Pomacentridae | Planktivore | Diurnal | Site attached |
| *Chromis atripectoralis* | Pomacentridae | Planktivore | Diurnal | Site attached |
| *Chromis iomelas* | Pomacentridae | Planktivore | Diurnal | Site attached |
| *Chromis margaritifer* | Pomacentridae | Planktivore | Diurnal | Site attached |
| *Dascyllus flavicaudus* | Pomacentridae | Planktivore | Diurnal | Site attached |
| *Dascyllus trimaculatus* | Pomacentridae | Planktivore | Diurnal | Site attached |
| *Pomacentrus coelestis* | Pomacentridae | Omnivore | Diurnal | Site attached |
| *Stegastes nigricans* | Pomacentridae | Herbivore/detritivore | Diurnal | Site attached |
| *Chlorurus spilurus* | Scaridae | Omnivore | Diurnal | Mobile within reef |
| *Scarus chameleon* | Scaridae | Omnivore | Diurnal | Mobile within reef |
| *Scarus forsteni* | Scaridae | Omnivore | Diurnal | Mobile within reef |
| *Scarus globiceps* | Scaridae | Omnivore | Diurnal | Mobile within reef |
| *Scarus psittacus* | Scaridae | Omnivore | Diurnal | Mobile within reef |
| *Scarus schlegeli* | Scaridae | Omnivore | Diurnal | Mobile within reef |
| *Dendrochirus biocellatus* | Scorpaenidae | Piscivore | Nocturnal | Site attached |
| *Cephalopholis argus* | Serranidae | Piscivore | Cathemeral | Site attached |
| *Epinephelus merra* | Serranidae | Piscivore | Cathemeral | Mobile within reef |
| *Epinephelus tauvina* | Serranidae | Piscivore | Cathemeral | Mobile within reef |
| *Pseudanthias mooreanus* | Serranidae | Planktivore | Diurnal | Site attached |
| *Sphyraena forsteri* | Sphyraenidae | Piscivore | Nocturnal | Mobile across reefs |
| *Corythoichthys flavofasciatus* | Syngnathidae | Invertivore (mobile) | Diurnal | Site attached |
| *Canthigaster bennetti* | Tetraodontidae | Omnivore | Diurnal | Site attached |
| *Canthigaster solandri* | Tetraodontidae | Omnivore | Diurnal | Site attached |

**
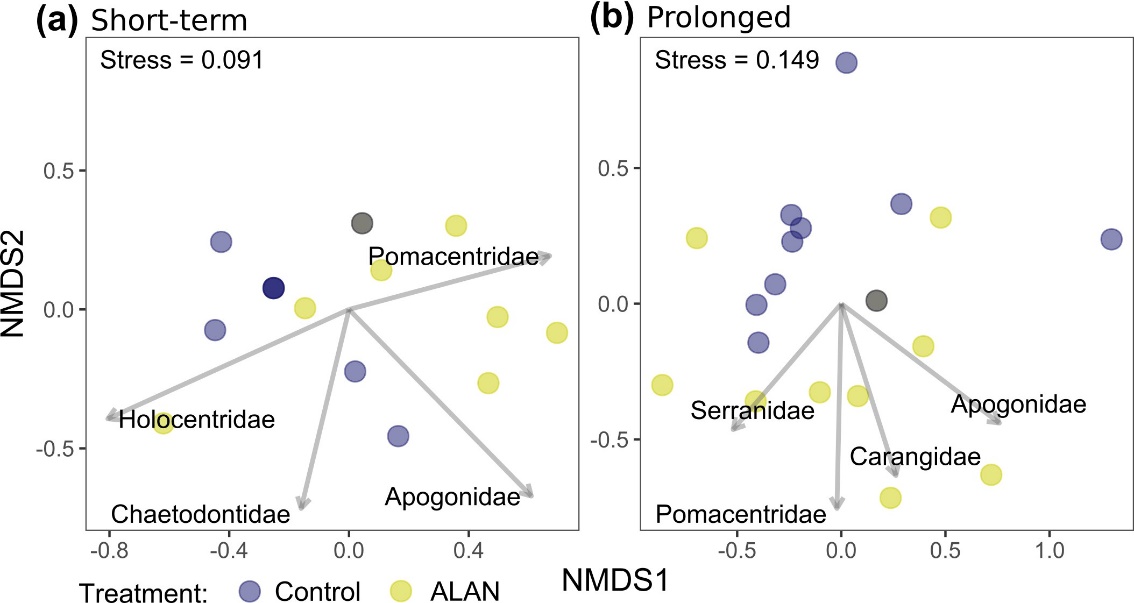
**

**Figure S2**. Non-metric multidimensional scaling (NMDS) ordinations displaying the variation in the composition of taxonomic families in the nighttime fish community after **(a)** short-term and **(b)** prolonged exposure to ALAN (yellow) and control conditions (blue). Fitted labelled vectors show the most influential families that are driving the spread of data. Data points represent individual sites (n = 16 for short-term and n = 20 for prolonged); darker points indicate overlapping data of the same treatment while mixed colours indicate overlapping data of different treatments. The stress values obtained indicate good representations of the distribution of taxonomic families.

**Table S3.** SIMPER analysis output presenting the taxonomic families ordered by greatest contribution toward dissimilarities between nighttime communities exposed to **(a)** short-term and **(b)** prolonged ALAN vs control conditions.

|  | Average presence | |  |  |
| --- | --- | --- | --- | --- |
| **(a) Short-term** | Control | ALAN | Contribution % | Cumulative % |
| Holocentridae | 0.88 | 0.37 | 31 | 31 |
| Apogonidae | 0.25 | 0.63 | 25 | 56 |
| Serranidae | 0.13 | 0.38 | 15 | 71 |
| Pomacentridae | 2.00 | 2.38 | 14 | 85 |
| Chaetodontidae | 0.13 | 0.13 | 7 | 92 |
| Tetraodontidae | 0.13 | 0.00 | 4 | 96 |
| Acanthuridae | 0.00 | 0.13 | 4 | 100 |
| **(b) Prolonged** |  |  |  |  |
| Pomacentridae | 1.60 | 2.60 | 21 | 21 |
| Apogonidae | 0.40 | 1.20 | 21 | 42 |
| Holocentridae | 0.70 | 0.90 | 19 | 61 |
| Serranidae | 0.30 | 0.50 | 11 | 72 |
| Mullidae | 0.20 | 0.40 | 8 | 80 |
| Lutjanidae | 0.00 | 0.30 | 6 | 86 |
| Acanthuridae | 0.00 | 0.20 | 4 | 90 |
| Carangidae | 0.00 | 0.20 | 3 | 93 |
| Tetraodontidae | 0.10 | 0.10 | 3 | 96 |
| Balistidae | 0.00 | 0.10 | 2 | 98 |
| Chaetodontidae | 0.00 | 0.10 | 1 | 99 |
| Ostraciidae | 0.00 | 0.10 | 1 | 100 |


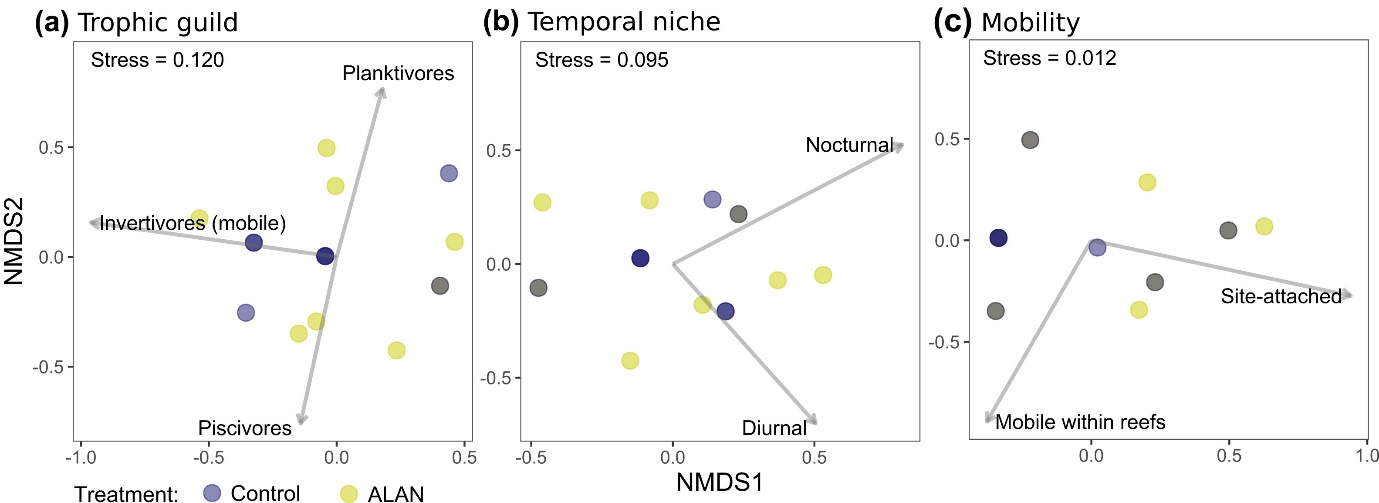


**Figure S3**. Non-metric multidimensional scaling (NMDS) ordinations displaying the variation in the composition of **(a)** trophic guilds, **(b)** temporal niches and **(c)** mobility types present in nighttime reef fish communities exposed to short-term ALAN (yellow) and control conditions (blue). Fitted labelled vectors show the most influential traits that are driving the spread of data. Data points represent individual sites (n = 16); darker points indicate overlapping data of the same treatment while mixed colours indicate overlapping data of different treatments. The stress values obtained indicate good representations of the distribution of trophic guilds, temporal niches and mobility types.

**Table S4.** SIMPER analysis output presenting traits within the functional groups: **(a)** trophic guild, **(b)** temporal niche and **(c)** mobility type, ordered by greatest contribution toward dissimilarities between nighttime communities exposed to short-term ALAN vs control conditions.

|  | Average presence | |  |  |
| --- | --- | --- | --- | --- |
| **(a) Trophic guild** | Control | ALAN | Contribution % | Cumulative % |
| Invertivores (mobile) | 1.13 | 0.88 | 44 | 44 |
| Piscivores | 0.13 | 0.38 | 19 | 63 |
| Omnivores | 0.13 | 0.38 | 18 | 81 |
| Planktivores | 2.13 | 2.25 | 14 | 95 |
| Herbivores + detritivores | 0.00 | 0.13 | 5 | 100 |
| Invertivores (sessile) | 0.00 | 0.00 | 0 | 100 |
| **(b) Temporal niche** |  |  |  |  |
| Nocturnal | 1.13 | 1.00 | 47 | 47 |
| Diurnal | 2.25 | 2.63 | 31 | 78 |
| Cathemeral | 0.13 | 0.38 | 22 | 100 |
| **(c) Mobility** |  |  |  |  |
| Site attached | 2.63 | 3.25 | 58 | 58 |
| Mobile within a reef | 0.88 | 0.75 | 42 | 100 |
| Mobile across reefs | 0.00 | 0.00 | 0 | 100 |

**References**

Froese, R., & Pauly, D. (2022). Fishbase, a global information system on fishes. World Wide Web electronic publication. http://www.fishbase.org

Mouillot, D., Bellwood, D.R., Baraloto, C., Chave, J., Galzin, R., et al. (2013) Rare species support vulnerable functions in high-diversity ecosystems. PLOS Biology, 11(5), e1001569. https://doi.org/10.1371/journal.pbio.1001569

Mouillot, D., Villéger, S., Parravicini, V., Kulbicki, M., Arias-González, J.E., Bender, M., Chabanet, P., Floeter, S.R., Friedlander, A., Vigliola, L., & Bellwood, D.R. (2014). Functional over-redundancy and high functional vulnerability in global fish faunas on tropical reefs. Proceedings of the National Academy of Sciences 111, 13757–13762. https://doi.org/10.1073/pnas.1317625111

Parravicini, V., Bender, M.G., Villéger, S., Leprieur, F., Pellissier, L., Donati, F.G.A., Floeter, S.R., Rezende, E.L., Mouillot, D., & Kulbicki, M. (2021). Coral reef fishes reveal strong divergence in the prevalence of traits along the global diversity gradient. Proceedings of the Royal Society B: Biological Sciences 288, 20211712. https://doi.org/10.1098/rspb.2021.1712
